# Improving the Transparency of Prognosis Research: The Role of Reporting, Data Sharing, Registration, and Protocols

**DOI:** 10.1371/journal.pmed.1001671

**Published:** 2014-07-08

**Authors:** George Peat, Richard D. Riley, Peter Croft, Katherine I. Morley, Panayiotis A. Kyzas, Karel G. M. Moons, Pablo Perel, Ewout W. Steyerberg, Sara Schroter, Douglas G. Altman, Harry Hemingway, for the PROGRESS Group

**Affiliations:** 1 Arthritis Research UK Primary Care Research Centre, Research Institute for Primary Care & Health Sciences, Keele University, Keele, Staffordshire, United Kingdom; 2 School of Health and Population Sciences, University of Birmingham, United Kingdom; 3 Arthritis Research UK Primary Care Research Centre, Research Institute for Primary Care & Health Sciences, Keele University, Keele, Staffordshire, United Kingdom; 4 Department of Epidemiology and Public Health, University College London, London, United Kingdom; 5 Centre for Molecular, Environmental, Genetic and Analytic Epidemiology, The University of Melbourne, Victoria, Australia; 6 Department of Oral and Maxillofacial Surgery, North Manchester General Hospital, Pennine Acute NHS Trust, Manchester, United Kingdom; 7 Julius Center for Health Sciences and Primary Care, UMC Utrecht, Utrecht, Netherlands; 8 London School of Hygiene & Tropical Medicine, London, United Kingdom; 9 Department of Public Health, Erasmus MC, Rotterdam, Netherlands; 10 BMJ, London, United Kingdom; 11 Centre for Statistics in Medicine, University of Oxford, Wolfson College Annexe, Oxford, United Kingdom; 12 Department of Epidemiology and Public Health and Director of the Farr Institute of Health Informatics Research at UCL Partners, London, United Kingdom

## Abstract

George Peat and colleagues review and discuss current approaches to transparency and published debates and concerns about efforts to standardize prognosis research practice, and make five recommendations.

*Please see later in the article for the Editors' Summary*

**Summary Points**

- Prognosis research is concerned with predicting outcomes to make health care more effective. It has a crucial role to play in clinical and policy decision-making.
- The quality of much prognosis research is poor, evidenced by incomplete reporting, poor data sharing, incomplete registrations, and absent study protocols.
- Initiatives to improve transparency in trials include reporting guidelines, data pooling, registers, and journal requirements for protocols. Prognosis research could be transformed by similar initiatives.
- Routine registration of all prognostic studies, linked to an accessible study protocol using agreed reporting guidelines, would improve transparency and promote data sharing.
- Concern about applying transparency methods to observational research could be resolved by flexibility to update date-stamped protocols during prognosis studies.

## Introduction

Predicting the future is big business in health care and medical research. Prognosis research focuses on the risk of future outcomes among individuals with a given disease or health condition and how this can be used to make care more effective [1]. It spans a wide spectrum of activity using study designs from both sides of the observational-experimental divide ([1]–[4]), ranging from the discovery of novel markers of prognosis [5], via multivariable risk prediction modelling [6], to randomised clinical trials (RCTs) of the impact of introducing prognosis tools into clinical practice [7]. The information on which it can draw is expanding rapidly, as databases linking health care data to genetic, biological, psychological, and social measures become widely available.

Recent examples of the significance of prognosis research include the relevance of international contrasts in breast cancer mortality for national policies of cancer registration and service organisation [8]; disparities between UK and Sweden in short-term survival following acute myocardial infarction and large differences in the uptake of primary percutaneous coronary intervention and beta-blockers [9]; the less favourable cost-effectiveness of treatment when evaluated using data on prognosis and patterns of use in “real-world” clinical practice as opposed to narrow trials data [10]; and the recent call to better understand the natural history of small pulmonary emboli detected by computed tomography pulmonary angiography [11], which reflects wider concern over the prognostic relevance of incidental findings from new diagnostic technologies.

The consequences of poor prognosis research for policy and practice are substantial too. The high-profile retraction of publications reporting better prediction of cancer outcomes by novel gene expression profiling [12] came only after erroneous findings had been extensively cited in the medical literature and used to justify initiating three clinical trials. A lack of consistently strong data management, lack of independent confirmation of the initial discovery, failure to lock down the specific test methods, and inadequate validation of the prognostic test prior to commencing clinical trials, all contributed to this failure [13]. Whistle-blowers responsible for identifying the failure advocated sharing of datasets and details of analysis to enable rapid replication [14]. Such cases may be exceptional, but the quality and rigor of much prognosis research has been more widely questioned [15]. Systematic reviews in prognostic factor research have failed to reach robust conclusions, citing publication bias, selective reporting of results, inadequate measurement of confounding, bias in study design, and small sample sizes within primary studies [16]–[18]. Holmes and colleagues [19], in their systematic review and meta-analysis of *CYP2C19* genotype as a predictor of differential response to clopidogrel, found many “treatment only” studies ill-suited to evaluating differential treatment response, and evidence of publication bias in small studies. The conclusion of the review challenged US Food and Drug Administration recommendations for genotyping to be considered before prescribing clopidogrel.

Prognosis research using cohort studies is no different to RCTs in requiring rigorously peer-reviewed protocols on which to base funding and ethical approval. Yet the need for transparency measures in prognosis research, including registration and pre-published protocols, similar to those expected and encouraged for RCTs by funders and journal editors and bodies such as the World Health Organization, is not widely recognised or accepted, despite recent calls for reform of journal and funders' policies towards transparency in observational research [20],[21].

Our ambition is to highlight why a concern for the transparency of prognosis research is an urgent and important priority for the public, patients, and medical and health care and research communities. We base this ambition on the unique perspective of prognosis research spanning observational studies and clinical trials, its rapidly expanding importance for clinical decision-making and health policy, and the demonstrable consequences of poor quality studies. In this paper we consider the arguments that have been made for and against measures to improve transparency, and emphasise practical measures to achieve better transparency for prognosis studies. Our appeal is principally to the research community, although we draw attention to the broader debate and changing policy environment. We consider the case for action on four issues: complete and accurate peer-reviewed reporting of study findings, facilitation of data sharing, study registration, and publicly accessible study protocols. We make recommendations for improving the transparency of prognosis research.

## Complete and Accurate Reporting

Transparent and complete study reporting is important because decision making in clinical practice and policy relies on bias-free evidence, and study generalizability and usefulness for meta-analysis and decision modelling rely on completeness and detail [22].

Reporting deficiencies in published prognosis studies are common [23]–[25], with omission of rudimentary results and methods such as the number of patients and events, or the number of prognostic candidate factors examined. Simon [26] comments that prognosis literature “is probably cluttered with ‘false-positive’ studies that would not have been submitted or published if the results had come out differently,” although this view has been challenged [27]. Within-study selectivity is probably worse than for trials because reports focus on prognostic factors, endpoints, threshold levels, and subgroups that produce “favourable” results.

For observational studies and some types of prognosis studies, reporting guidelines exist, but are not widely used. Relevant for studies that investigate whether specific factors influence patient outcome (“prognostic factor studies”) are published guidelines for tumour marker research (REporting recommendations for tumor MARKer prognostic studies [REMARK] [28]), which cover all components of the study process from hypotheses to results and limitations. The importance of clear reporting for particular study designs is emphasized in reporting guidelines for studies designed to develop models that predict an individual's likelihood of a particular outcome (“prognostic model studies”) (TRIPOD: http://blogs.bmj.com/bmj/2011/08/03/gary-collins-opening-up-multivariable-prediction-models/) and for studies that use electronic health records (RECORD [29]).

## Data Sharing

Data sharing makes data collected for one purpose accessible to other investigators, including data curation activities such as documentation of meta-data (data about data), harmonisation procedures, and tools to support the accessibility of datasets. All these activities need to be conducted within a strong governance framework. Important examples include large scale biobank cohorts [30] and e-health record linkages [31].

Patient concern about regulation of use of their data for research co-exists with public interest in ensuring maximal benefits for the public from such data. However, realising this goal on a large scale requires navigation through consent and confidentiality; standards and frameworks for data formatting; planning and management of original data; funding and incentives for data archiving and intellectual property; and communication between custodians and secondary users of data [32]. The exact governance, consent, and access arrangements that are in place will determine the nature and extent of data sharing, and funders' policies in this area continue to evolve (http://www.wellcome.ac.uk/About-us/Policy/Spotlight-issues/Data-sharing/EAGDA/index.htm). Current options include the negotiation of secure, remote access to anonymised data (e.g., access to Swedish registry data by Chung et al. [9]), the release of copies of whole or selected parts of anonymised datasets subject to data sharing agreements (e.g., Osteoarthritis Initiative, http://oai.epi-ucsf.org/datarelease/), or the preference for researchers to come and analyse the data at the host centre (as per Danish National Birth Cohort (http://www.ssi.dk/English/RandD/Research%20areas/Epidemiology/DNBC/For%20researchers/Conditions%20for%20access%20to%20data.aspx)).

Data sharing for prognosis research is uncommon and most meta-analyses do not use individual patient data (IPD). Several reports have considered pros and cons [32],[33]; Altman and colleagues [34] conclude that obtaining IPD was “long, expensive, and … laborious.” However, IPD meta-analyses of prognosis studies are now emerging [35]; one recent review found 48 published IPD meta-analyses of prognostic factors [36]. In traumatic brain injury, researchers initiated IMPACT (International Mission for Prognosis and Analysis of Clinical Trials) and meta-analysed IPD from 11 studies including 9,205 patients [37].

Registration and data sharing in trials has enabled adequately powered IPD meta-analyses to examine predictors of differential treatment response and development of prognostic models using IPD from multiple trials or prognosis studies. Such initiatives show that data sharing and international collaboration is possible and productive in prognosis research, and should encourage others as the volume of information available for analysis on a large-scale from health care and genetic databases continues its rapid expansion.

Funders of prognosis research should require data sharing with appropriate governance. This requirement is increasingly occurring as applicants are routinely asked to specify how new data is to be shared and made accessible (e.g., www.mrc.ac.uk/Fundingopportunities/Grants/Researchgrant/index.html).

Vandenbroucke [27] has raised concerns about the double-edged nature of data sharing (“re-analysis is a superb tactic to delay regulation” of treatments or exposures found to be harmful, for example). Against this concern is trials literature that long ago demonstrated that timely meta-analysis would have produced earlier results [38] and IPD would logically extend that capacity. Integrated data from prognosis studies, facilitated by standardisation of methods and measures, could deliver timely, practical results to support clinical decisions. One-off prognosis studies will rarely drive clinical practice.

## Study Registration

Study registers hold an internationally agreed minimum amount of information about research studies in a publicly available database. Trials registration was proposed to meet ethical obligations and reduce bias through better design, encouragement to publish, and full outcome reporting [39]. Several registries meet the International Committee of Medical Journal Editors (ICMJE) requirements, and advance registration is a condition for trial publication in many but not most leading journals [40],[41]. However some claimed benefits have been challenged [42].

Two decades ago, Dickersin proposed that “registration of clinical trials, *and perhaps other types of studies*, is …(where) …scientific community should move” [43]. Observational studies now constitute a small but increasing minority of registered studies on ClinicalTrials.gov (15% in 2006 to 21% in 2012); fewer than 1% are indexed using the terms “prognosis” or “prognostic.” Recent papers have considered the general case for registration of observational studies [44]. Others have questioned uncritical application of arguments and policies derived from clinical trials to observational studies [45].

### Reducing Publication and Reporting Bias

Bias due to non-publication and within-study selective reporting exists in prognosis research. Kyzas and colleagues [23] found fewer than 2% of 1,915 articles on cancer prognostic factors contained no positive findings at all. Registration aligns with the need, recognised by proponents and opponents of registration alike, to abandon the culture of uncontrolled questing in datasets and reliance on significance testing to find “positive” study results. Given evidence about selective non-publication and reporting of prognosis studies, and unnecessary duplication of effort, study registration should help address these issues.

Some critics suggest many unpublished studies may simply be “fatally flawed… [or] of little consequence” [46], with registration simply uncovering more small, single-centre studies. They highlight the unpredictable effects of including unpublished trials in meta-analyses. The counterargument is that advance registration permits evaluation of these effects, even though it may not in itself prevent bias.

### Evaluation Versus Discovery, and the “Universe of All Ideas”

Vandenbroucke [47] has distinguished “evaluation” from “discovery.” Evaluation provides a single chance, optimally in a randomised trial, to test an intervention that might affect many lives, and needs to be highly regulated. Discovery pursues novel analyses as ideas develop during a particular study, which excessive regulation would hamper.

Prognosis research, however, blurs this distinction—assessment of outcomes is related to a wide universe of potential prognostic factors, and needs a simple framework for describing research planned and in progress. The need to account for all possible analyses is underlined by the rapid expansion of studies using large e-health databases from clinical practice.

Having a comprehensive register of ideas allows assessment of the universe of studies from which published results appear. Lash [42] and others argue that registration of whole databases, rather than individual studies, is the preferable route. But Thomas and Peterson [48] instead concluded that the potential gains from registering analysis plans (guarding against data dredging, reducing duplication) outweigh concerns (analyses dismissed if not pre-specified). They argue that analysis plans can be flexible enough to incorporate subsequent findings, and be revisited if there is a compelling reason.

### Evidence for the Benefits of Registration in Randomised Clinical Trials

Evidence about the effects of trial registration is limited. Reporting per guidelines is more complete for registered RCTs, but fewer than half are adequately registered. Registration does not prevent selective reporting altogether, but does help its identification. Van Enst and colleagues [49] found that, out of 210 Cochrane reviews of trials, only 80 had searched registries and 28 used them to identify additional trials.

This finding suggests trial registration is evolving and incomplete rather than misguided, and contradicts concerns that researchers might register every idea imaginable to claim territory and deter competition [45].

### Costs of Registration to Researchers

Costs and time needed to register are potential disadvantages for less well-resourced researchers, hindering rapid production of evidence or other “quirky, brilliant work that is not enterprise-driven” [45]. However fees are either non-existent (e.g., ClinicalTrials.gov) or reasonable (UK£210 plus VAT for International Standard Randomised Controlled Trial Number [ISRCTN]), and the level of detail needed to meet the main purpose of informing the scientific community of a study's existence does not impose a great burden on researchers.

### Content of Registration

Williams and colleagues [44] highlight how observational studies, including prognosis studies, can be added to an existing trial register (ClinicalTrials.gov), although it is acknowledged that these registries are likely to provide a better fit for new studies with prospective data collection [44] and that they are more cumbersome and challenging for prognosis studies based on re-analysis of existing datasets. Nevertheless, whole databases and patient registries can also be included [50], together with their primary outcome measurements and links to further information of particular importance for prognosis research, e.g., baseline variables. Table 1 outlines elements of a prognosis study or dataset that may be important to consider for registration, protocols, reporting, and data sharing. The WHO 20-item Trial Data Set is largely contained in these.

**Table 1:** Elements to consider including in registration, protocols, reporting, and data sharing^a^.

Stage	Elements
***Governance***	Title^b^; version^b^; date^b^; principal investigator(s)^b^; contacts^b^; sponsors^b^; institutional review board/ethics committee approval^b^; funding source^b^
***Rationale***	Translational gap or other scientific uncertainty being addressed
***Objective(s)***	Type of prognosis research, e.g., according to PROGRESS framework [1]:(i) the course of health related conditions in the context of the nature and quality of current care (fundamental prognosis research);(ii) specific factors (such as biomarkers) that are associated with prognosis (prognostic factor research);(iii) the development, validation, and impact of statistical models that predict individual risk of a future outcome (prognostic model research);(iv) the use of prognostic information to help tailor treatment decisions to an individual or group of individuals with similar characteristics (stratified medicine research)
***Study design***	Design type (e.g., randomised trial, prospective cohort, utilisation of existing database, systematic review)Target population, eligibility criteria^b^, startpoint, clinical setting^b^Disease/health condition phenotyping^b^;Treatments used;Factors of interest (e.g., definitions, timing, and methods of measurement);Sample size (e.g., rationale, expected number of events)^b^;Primary and secondary endpoints (e.g., definitions, timing, and methods of measurement)^b^.Cross reference to study registration, protocol, report(s), data sharing policy
***Statistical methods***	Statistical analysis techniques (e.g., logistic, survival regression)Strategy for including multiple variables (e.g., model selection choice);Dealing with missing data (e.g., complete case analysis versus multiple imputation);Handling of continuous variables (e.g., variable transformation, modelling non-linear trends, choice of threshold level if any);Choice of subgroup analyses;Measures to assess model performance (e.g., internal validation; external validation criterion)
***Results***	Document how results will be presented, including:Descriptive results (e.g., number of patients and events);Kaplan-Meier curves;Univariable and multivariable results for each factor and outcome;Effect estimates (e.g., odds ratios, hazard ratios) and confidence intervals;Prognostic model parameter estimatesSummary statistics and graphs for model performance (e.g., calibration and discrimination);Estimate (with confidence interval) of interaction between factor and treatment effect

a Stages mirror the structure of research publications; registration tends to have fewer elements.

b Overlap with WHO Trial Registration Dataset v1.2.1. and ClinicalTrials.gov.

How far to require pre-specification of all analyses of prognosis data? One advantage of a register (as compared with journal publication of a protocol) is that it can be updated to incorporate new information or planned objectives, and the date of updating archived. New registers designed for prognosis research, starting with studies planning prospective data collection, may eventually provide the best approach, although the merits of cross-disease, cross-research-question registries need to be considered.

### Timing of Registration

**Figure 1:**
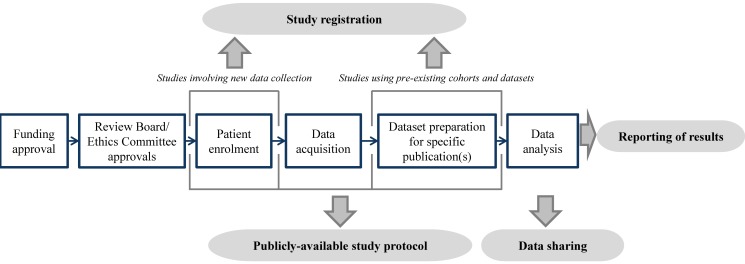
Illustration of the timing of prognosis study registration, protocol publication, data sharing, and reporting.

Given that registers should provide a view of current prognosis research activity, registration should occur early—prior to data acquisition for planned cohort studies, and prior to sampling or analysis for prognosis studies of pre-existing cohorts or datasets. Figure 1 illustrates optimal points. Sørenson and Rothman [45] point out that this timing does not preclude bias in the selection of pre-specified analyses. However, it does allow the pool of intended analyses from which publications are drawn, and the selectivity of the pre-specified questions, to be examined.

## Publicly Accessible Study Protocol

### The Case for Protocols

Protocols describe the rationale, objectives, design, methodology, statistical considerations, and organisation of a study and they present a research plan made before the conduct of the study. Compared to study registers, protocols contain more detail, particularly about study design and analysis plans. There is a strong link between what appears in a protocol and what is reported in research publications, which may mirror reporting guidelines (e.g., REMARK [28]) or guidelines for data collection and curation (e.g., MIAME [51] for gene expression studies).

Most reports from prognosis studies do not refer to a protocol. Prognosis studies are often piecemeal and opportunistic with no peer reviewed funding or protocols. Most do not explicitly build on previous findings, are often too small to answer the research question, and typically do not provide sample size calculations [24],[25]. Yet funders, ethical committees, and dataset curators require rigorous peer-reviewed protocols before approving prognosis studies. In this way they ensure that researchers make their work amenable to comment and have to critically consider key elements in design prior to data acquisition or analysis, and this helps drive originality and quality in prognosis research. The expectation therefore is that all prognosis studies should have a protocol, written before data acquisition in new cohorts and before sampling and analysis of established datasets, timings that align with our recommendations for registration. Electronic records of study protocols, including date stamping, will enable documentation of the conception of a planned analysis (e.g., pre-, ad-, or post-hoc).

However, a prognosis research protocol cannot be a rigid blueprint; it is neither possible nor desirable to pre-specify all analyses. A protocol may state that the goal is exploration, or that modification or additional analyses may be needed in the light of new findings. Even highly exploratory or data-driven analyses to discover new prognostic factors can have a protocol, albeit in broad terms. A possible exception would be analyses of already-collected data aimed at immediate replication of important or controversial discoveries where ideally the protocol from the work that is being replicated would be available. Exploration of pre-existing and readily available data is an accepted, valuable part of epidemiologic research practice. This activity can, in our view, co-exist with a move towards greater transparency for all prognosis studies; if a study is exploratory this can be stated.

### Benefits of an Accessible Protocol

Evidence is accruing for the benefits of making a pre-specified protocol publicly available. It allows scientific peers in principle to replicate the study; easier identification of, and access to, full study details; and more opportunities for collaboration including systematic reviews and IPD meta-analyses. The existence of a publicly available protocol enhances the credibility of the research, permits authors to cross-reference detail to the protocol, and provides a basis for defending “negative” or statistically non-significant results to editors and reviewers. Experience with trials is that an accessible protocol, together with registration, facilitates systematic evaluation of selective non-publication and reporting [39]. An accessible protocol can be compared against final reports to identify potential reporting bias, and allows the researcher to defend choice of outcomes.

### Making Prognosis Research Study Protocols Accessible

Stand-alone publication of protocols in journals is one option and creative new models of publishing, including in-principle acceptance of papers arising from pre-registered and reviewed protocols [52], may provide further encouragement to researchers. However, the number of study protocols available by this route is currently limited, as is the scope for updating protocols.

The other option is that protocols (whether or not they are journal publications) are made accessible by linkage to registration (e.g., using the “Detailed Description” field of ClinicalTrials.gov or as an attachment under “More Information”). Dated protocol changes and their rationale can then be documented (e.g., via the “History of Changes” link in ClinicalTrials.gov). Date-stamped copies of protocols can also be uploaded onto researchers' host institution study website or research funders' websites, and linked to registration.

Pre-specification by this route can thus embrace approaches that emerge during analysis—this applies to both observational studies and RCTs [48]. Reasons for protocol changes can be specified initially and at updating, indicating whether additional analyses were data-driven or not. Reporting these, in the context of the original protocol, helps better understand and assess the results. The REMARK reporting profile encourages broad analysis strategies to be pre-specified at registration, but recognises other data-driven analyses may emerge [28]. As a minimum, journal editors could require protocol availability at the time of the publication of research results. Ideally, however, accessibility should align with registration (Figure 1). Regardless of whether and where the protocol is made publicly available or accessible, it should be “date stamped” for future reference.

## Conclusions and Recommendations

We have argued that the quality of prognosis research could be substantially improved by the adoption and promotion of straightforward methods to improve transparency. The range of ethical and scientific benefits that should accrue from the adoption of transparency measures outlined in this report extends beyond just those considered here and Table 2 provides a summary. Our recommendations (summarised in Table 3) are designed to encourage prognosis researchers to realise these benefits.

**Table 2:** Potential benefits of study registration, protocol publication, better study reporting, and data sharing of prognosis research studies.

Potential Benefit	Registration	Protocols	Reporting	Sharing
**Ethical**				
Respect the investigator-participant covenant to generate new, publicly accessible biomedical knowledge of potential value to future patients	X	X	X	X
Facilitate monitoring and accountability in relation to global standards for ethical research, including informed consent	X	X	X	
Cost-effective use of public money	X	X	X	X
**Scientific**				
Improve the quality and reliability of evidence from prognosis research, (and thereby enhance impact on health and health care)	X	X	X	X
Help accelerate knowledge creation through easier identification of and access to full study details, including data, in order to increase opportunities for collaboration including systematic reviews and meta-analysis	X	X	X	X
Answer research questions only possible through collaboration				X
Reduce unnecessary duplication of invested research resources through awareness of existing studies	X	X		
Establish intellectual property		X		
Provide a denominator against which publication bias can be assessed	X	X		
Provide means for identification and prevention of biased under-reporting or over-reporting of research		X	X	
Involve patients in studies, including enrolment	X	X		
Peer review of protocols to improve study quality and refine methods		X		
Methodological issues sufficiently detailed to, in principle, allow study replication (details not always allowable in published reports)		X		

**Table 3:** Recommendations.

Number	Recommendation
**1**	**Full study reporting through use of guidelines**
	• Extended versions of REMARK guidelines to be developed for prognosis research
**2**	**Facilitate and expect data sharing**
	• Promote standardised case definitions and outcome measures
	• Create culture where data sharing is a positive achievement
**3**	**Routine registration of all prognosis studies using existing registers**
	• Establish minimal dataset (startpoint; list of candidate factors)
	• Broad analysis plan included, allowing for updates
	• Register before data acquisition in new cohorts and before sampling and analysis in established datasets
**4**	**Protocols for all prognosis studies made public**
	• Encourage early public accessibility through linkage to registration record or journal publication
	• Original protocol and amendments should be available when results submitted for publication
	• Align standard elements of protocol with guidelines for publication and core registration dataset
	• Date stamp electronic protocols—update with revision
**5**	**Promote systematic development and evaluation of methods and value of transparency**
	• Objective basis for change and improvement
	• Systematic evaluation of adoption of transparency measures and their impact

Complete and accurate reporting of all components of a prognosis study could be achieved by application of existing but underused published reporting guidelines (recommendation 1). We propose that extended versions of the existing REMARK guidelines for tumour marker studies should be developed for application to all prognosis research.

Data sharing for prognosis research is still uncommon, but there are good examples of the benefits of accessible and combined datasets, notably for IPD meta-analyses. We recommend that data sharing should be the normal expectation in prognosis research, and that standardisation of measures in prognosis studies should be promoted (recommendation 2).

Registration of RCTs has required changes not only in editorial policy, legislation, and regulation, but also in research culture, and further improvements are still needed [53] We consider the lack of an ideal registration culture in trials is not an argument against registration of prognosis studies. Registration is a simple low-cost initiative that we recommend for all prognosis research studies (recommendation 3). An agreed minimal dataset should be developed for this purpose.

Study protocols extend the detail available in registers. It seems reasonable to encourage protocols for all prognosis research (recommendation 4) since research funders and many large cohorts require them *de facto*. We also recommend early accessibility and public availability of prognosis research protocols. Journal publication is an important method of making protocols readily available, and there should be a minimum requirement for a protocol to be accessible at the time of publication of the results of the study. However linking accessible time-stamped protocols to study registration would achieve accessibility for all registered studies and allow data analysis proposals to be updated during the evolution of a study whilst retaining the original protocol.

Finally transparency itself requires systematic approaches to developing methods to achieve improvement (for example, evidence-based consensus on core content of registration record for prognosis studies). We conclude also that there must be critical and systematic evaluation of the success of these methods in achieving the aims of transparency, namely better quality prognosis research, more efficient use of available data, and a research culture that can keep pace with rapidly expanding clinical and health care data in an era of greater patient involvement and public accountability (recommendation 5).

## Abbreviations

IPD: individual patient data
RCT: randomised clinical trial
